# Effect of Origin, Seed Coat Color, and Maturity Group on Seed Isoflavones in Diverse Soybean Germplasm

**DOI:** 10.3390/plants13131774

**Published:** 2024-06-27

**Authors:** Muhammad Azam, Shengrui Zhang, Jie Qi, Ahmed M. Abdelghany, Abdulwahab Saliu Shaibu, Yue Feng, Suprio Ghosh, Kwadwo Gyapong Agyenim-Boateng, Yitian Liu, Luming Yao, Jing Li, Bin Li, Biao Wang, Junming Sun

**Affiliations:** 1 School of Agriculture and Biology, Shanghai Jiao Tong University, Shanghai 200240, China; azaamuaf@gmail.com (M.A.); lmyao@sjtu.edu.cn (L.Y.); 2 The State Key Laboratory of Crop Gene Resources and Breeding, National Engineering Laboratory for Crop Molecular Breeding, Institute of Crop Sciences, Chinese Academy of Agricultural Sciences, 12 Zhongguancun South Street, Beijing 100081, China; zhangshengrui@caas.cn (S.Z.); qjycyz@gmail.com (J.Q.); asshuaibu.agr@buk.edu.ng (A.S.S.); yue.feng-5@syngentagroup.cn (Y.F.); fssuprio@gmail.com (S.G.); k.g.agyenim.boateng@gmail.com (K.G.A.-B.); 82101201051@caas.cn (Y.L.); lijing02@caas.cn (J.L.); libin02@caas.cn (B.L.); 3 Department of Agronomy, Bayero University, Kano 700001, Nigeria; ahmed.abdelghany@agr.dmu.edu.eg

**Keywords:** soybean, isoflavones, country of origin, maturity group, seed coat color, correlation

## Abstract

Soybeans are grown worldwide owing to their protein, oil, and beneficial bioactive compounds. Genetic and environmental factors influence soybean seed isoflavones. In the present study, we profiled the seed isoflavones in world diverse soybean germplasm grown in two locations over two years in China. Significant differences (*p* < 0.001) were observed between the accessions, accession origins, seed coat colors, and maturity groups for individual and total isoflavone (TIF) content. TIF content of the soybean accessions ranged from 677.25 μg g^−1^ to 5823.29 μg g^−1^, representing an 8-fold difference. USA soybean accessions showed the highest mean TIF content (3263.07 μg g^−1^), followed by Japan (2521.26 μg g^−1^). Soybean with black seed coat showed the highest (3236.08 μg g^−1^) TIF concentration. Furthermore, isoflavone levels were significantly higher in late-maturity groups. Correlation analysis revealed significant positive associations between individual and TIF content. Malonyldaidzin and malonylgenistin showed higher correlations with TIF content (*r* = 0.92 and *r* = 0.94, respectively). The soybean accessions identified as having high and stable TIF content can be utilized in the food and pharmaceutical industries and breeding programs to develop soybean varieties with enhanced isoflavone content.

## 1. Introduction

Soybean is an important food and feed crop in the world. It is a good source of high-quality vegetable protein, oil, and bioactive compounds such as fatty acids, carbohydrates, folates, tocopherols, and isoflavones [1,2]. The isoflavones found in soybean seeds have beneficial effects on human health, including the prevention and treatment of various types of cancers, metabolic syndromes (Type 2 diabetes and obesity), neurodegenerative disorders (Alzheimer’s and Parkinson’s), cardiovascular diseases, and osteoporosis [3,4,5,6,7]. Soybean seed isoflavones are categorized into aglycones, glycosides, acetylglycosides, and malonylglycosides. Malonylglycosides are the predominant form of isoflavones in soybean seeds [8,9,10]. Improving soybean seed quality is challenging due to the low genetic variation in soybean cultivars. Therefore, profiling seed isoflavones in diverse soybean germplasm is desirable, as it will provide new breeding materials to soybean breeders [11,12].

Soybean seed isoflavones are affected by both genetic and environmental factors [13,14,15]. Previous studies have reported that apart from the genetic and environmental factors, soybean origin, maturity group, and seed coat color significantly affect isoflavone accumulation in soybean seeds [16,17,18,19]. Soybean seeds display various colors, such as yellow, green, brown, purple-red, black, and bicolor [20,21]. Physiological and genetic studies have reported that different metabolite accumulations are responsible for the seed coat color variations in soybeans [22,23,24]. Previous studies have reported the influence of origin and seed coat color on soybean seed isoflavones [19,25], but these studies utilized few soybean accessions, which makes it quite challenging to analyze the impact of origin, maturity, and seed coat color on soybean seed isoflavones and to select high-quality germplasm. 

Introducing new germplasm is widely recognized as essential for achieving greater genetic diversity and enhancing breeding resources. Germplasm collections exhibit significant diversity in their origins and may provide valuable accessions that can be used in various crop breeding plans [16,19]. Therefore, profiling soybean seed isoflavones in a large germplasm collection across multiple locations for multiple years is needed to investigate the effect of origin, maturity group, and seed coat color on seed isoflavones and to identify high-quality soybean germplasm, as it will help soybean breeders to improve the nutritional aspects of soybean varieties.

Therefore, in the present study, 593 soybean accessions were grown across multiple locations for multiple years with the following objectives: (i) to profile the isoflavones in soybean germplasm of different origins and seed coat colors; (ii) to investigate the effect of maturity group (MG) on soybean seed isoflavones; (iii) to explore the relationships between individual isoflavone contents; and (iv) to identify soybean accessions having high and stable total isoflavone (TIF) content across different environments. This research will provide high-quality soybean accessions with higher TIF to the breeders to develop new cultivars, and it can also be utilized directly in the industry to meet consumers’ needs.

## 2. Results and Discussion

### 2.1. Natural Variation of Isoflavones in Diverse Soybean Germplasm

Individual and TIF content had significant variation (*p* < 0.001) among the soybean accessions (Supplementary Table S3). The mean TIF concentration ranged from 677.25–5823.29 μg g^−1^ with a mean of 2670.70 μg g^−1^ (Table 1). The highest levels of mean TIF content (5823.29 μg g^−1^) were observed in the accession WDD01618 from the USA, while the lowest levels of TIF content (677.25 μg g^−1^) were identified in the accession ZDD13152 from China. TIF content exhibited a significant 8-fold variation between the soybean accessions with the highest and lowest levels. These results showed much more variation compared to previous studies [8,9,19]. Six accessions were identified as having a mean TIF concentration > 5000 μg g^−1^, and two accessions had a mean TIF concentration < 1000 μg g^−1^. The six soybean accessions identified had higher TIF content than that of the novel soybean cultivar Zhonghuang 68 developed at the Institute of Crop Sciences, CAAS, Beijing, which is reported to have a high isoflavone content of 5135.86 μg g^−1^ [12] (Supplementary Table S4). Previous studies have identified malonylglycosides as the most abundant isoflavones [9,10]. In the present study, malonylglycosides (83.03%) are also identified as the major components, followed by glycosides (15.72%) and aglycones (0.84%). The accessions and years showed significant differences for all the isoflavone content (Supplementary Table S3). These results are in agreement with previous studies, which also reported that genetic and seasonal variabilities affect isoflavone accumulation in the soybean seed [13,26,27], but greater variations among soybean accessions for the individual and TIF content in the present study also confirm that accession genetic makeup had a pronounced effect on soybean seed isoflavone accumulation.

### 2.2. Effect of Origin on Soybean Seed Isoflavones

**Figure 1:**
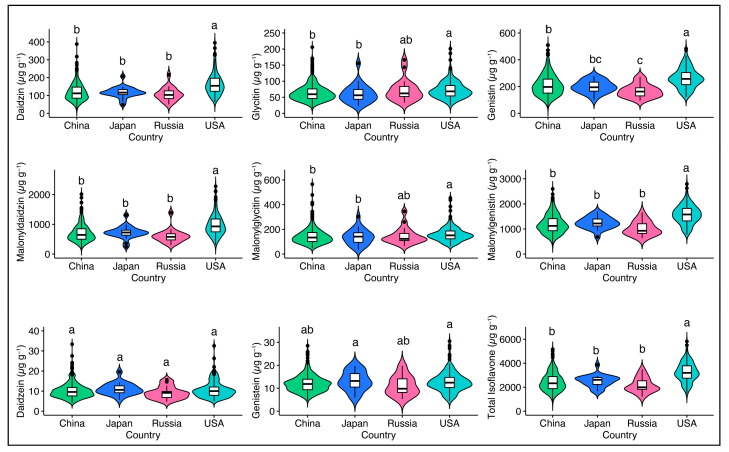
The levels of individual and total isoflavones in soybean accessions from different origins. Pairwise comparisons were conducted using a least significant difference (LSD) test. Lowercase letters (a, b, and c) denote statistically significant differences at the *p* < 0.05.

The countries of origin showed significant differences (*p* <0.001) for individual and TIF content except for daidzein (Supplementary Table S3). The TIF concentration ranged from 677.25–5170.84 μg g^−1^ in the Chinese accessions (391 accessions), 1675.28–3871.6 μg g^−1^ in the Japanese accessions (22 accessions), 1199.98–3492.86 μg g^−1^ in the Russian accessions (19 accessions), and 1462.19–5823.29 μg g^−1^ in the USA soybean accessions (161 accessions). The USA accessions had the mean TIF concentration (3263.07 μg g^−1^), followed by those from Japan (2521.26 μg g^−1^) and China (2459.55 μg g^−1^), while the Russian accessions had the mean TIF concentration (2169.67 μg g^−1^) (Figure 1). 

Furthermore, the USA soybean accessions showed higher concentrations of glycoside (daidzin (164.32 μg g^−1^), glycitin (73.29 μg g^−1^), genistin (263.2 μg g^−1^)) and malonylglycosides (malonyldaidzin (979.25 μg g^−1^), malonylglycitin (160.36 μg g^−1^), malonylgenistin (1574.46 μg g^−1^)) compared to other countries, while higher levels of aglycones (daidzein (11.11 μg g^−1^), genistein (13.16 μg g^−1^)) were observed in the Japanese soybean accessions (Figure 1). These results showed great variations in isoflavone content compared to previous studies investigating the effect of origin on soybean seed isoflavones [19,28,29]. The large variations in TIF content compared to previous studies might be attributed to the large number of diverse soybean accessions employed in the present study. The significant differences in individual and TIF content (Supplementary Table S3) show that country of origin significantly affected the accumulation of soybean seed isoflavones. The TIF concentrations of these soybean germplasms will vary if they were grown in their regions of origin. Our findings suggest that the accessions developed in the USA have higher isoflavones, and the high-quality USA soybean accessions with higher TIF content can be included in the breeding program. 

### 2.3. Seed Coat Color Significantly Influenced the Soybean Seed Isoflavones

**Figure 2:**
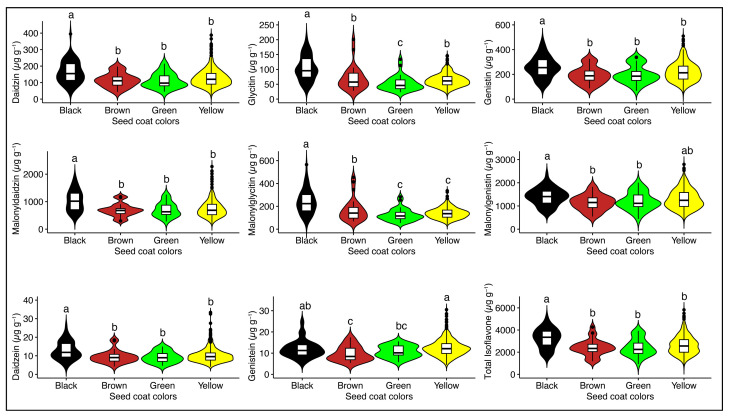
The levels of individual and total isoflavones in soybean accessions of different seed coat colors. Pairwise comparisons were conducted using a least significant difference (LSD) test. Lowercase letters (a, b, and c) denote statistically significant differences at *p* < 0.05.

Soybean seed coat colors showed significant differences (*p* < 0.001) for individual and total isoflavone content (Supplementary Table S3). Among soybean seed coat colors, higher mean levels of TIF content (3236.08 μg g^−1^) were observed in black coat color seeds, followed by yellow, green, and brown, having mean TIF contents of 2630.24 μg g^−1^, 2429.82 μg g^−1^, and 2409.64 μg g^−1^, respectively (Figure 2). These results showed great variation from the previous studies that investigated the influence of seed coat color on isoflavone contents [17,19,25].

Soybean isoflavones include aglycones, glycosides, acetylglycosides, and malonylglycosides. In the present study, malonylglycosides showed higher concentrations, followed by glycosides, and aglycones showed lower concentrations; a similar comparison was also reported in previous studies [8,10]. Furthermore, significant differences (*p* < 0.001) among seed coat colors for individual and total isoflavone content (Supplementary Table S3) also revealed that seed coat color has a pronounced effect on soybean seed isoflavone accumulation. Taken together, agronomic, genetic, and seasonal factors should be considered while growing soybean accessions for isoflavones, and high-quality black soybean seed accessions can be used in food, feed, and breeding according to consumers’ needs.

### 2.4. Effect of Maturity Group on Soybean Seed Isoflavones

**Figure 3:**
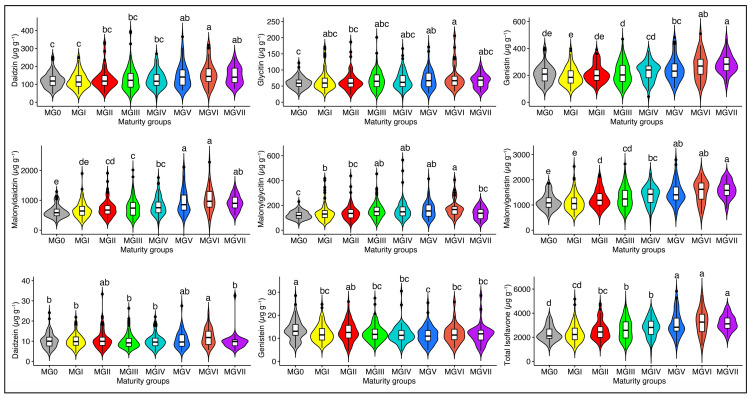
The levels of individual and total isoflavones in soybean accessions of different maturity groups [MGs]. Pairwise comparisons were conducted using a least significant difference (LSD) test. Lowercase letters (a, b, c, d, and e) denote statistically significant differences at *p* < 0.05.

Maturity groups had significant effects (*p* < 0.001) on the individual and TIF content (Supplementary Table S3). The soybean seed isoflavones change considerably with maturity. The isoflavones variations among the maturity groups might be attributed to the differences in the growing locations used for evaluation. The highest levels of mean TIF content were observed in MGVI (3275.42 μg g^−1^), followed by MGVII (3194.81 μg g^−1^) and MGV (3100.40 μg g^−1^), while the lowest levels of mean TIF content were observed in MG0 (2291.88 μg g^−1^). MGVI showed higher levels of daidzin (157.34 μg g^−1^), glycitin (78.08 μg g^−1^), malonyldaidzin (1030.68 μg g^−1^), and malonylglycitin (180.86 μg g^−1^), while MGVII showed higher levels of genistin (283.54 μg^−1^) and malonylgenistin (1608.36 μg^−1^) (Figure 3). These results agree with previous studies that reported higher levels of isoflavones in late-maturity groups [10,16]. Early-maturing soybean accessions experience reduced levels of individual and total isoflavone content when exposed to long days and higher solar radiation conditions in high-latitude regions during the seed filling period (R5–R8 stage) [10,30]. The low isoflavone content can also be attributed to the origin and distribution of early-maturing accessions in high-latitude regions [27,31]. Accessions that matured later showed a greater concentration of isoflavones because late-maturing genotypes were exposed to colder temperatures during seed filling [32].

Soybean accessions are categorized into several maturity groups according to their specific photoperiod (day length) needs. Maturity group is a significant agronomic trait that influences the quality of soybean seeds. The ANOVA in this study demonstrated that various MGs made varying contributions to the isoflavone contents in soybean seeds (Supplementary Table S3). These results are consistent with previous research showing the impact of MGs on soybean seed isoflavones [10,33]. The MGs in our study showed a pronounced effect on the soybean seed isoflavones, and the late MGs showed higher levels of the individual and total isoflavones regardless of the environment, as evident from the non-significant MG × year interaction (Supplementary Table S3). The MGs with higher TIF concentrations will facilitate the utilization and movement of the accessions within and out of the country of origin. 

### 2.5. Correlation, Heatmap, and Stability Analysis of Isoflavones

**Figure 4:**
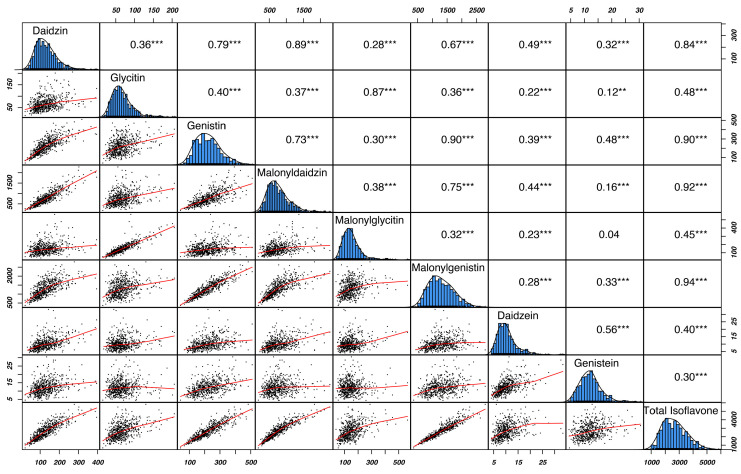
Correlation among individual and total isoflavone (TIF) content with significance levels at ** <0.01, and *** <0.001.

Correlation analysis between the individual and total isoflavone content clarifies how they are mutually associated and serve as a valuable tool in guiding breeding programs. Among the individual isoflavones, malonylgenistin and malonyldaidzin showed a higher correlation with TIF (*r* = 0.94 *** and *r* = 0.92 ***, respectively), while genistein and daidzein showed a lower correlation with TIF (Figure 4). Glycosides showed higher associations with their respective malonylglycosides, and positive correlations were observed between daidzin and malonyldaidzin (*r* = 0.89 ***), glycitin and malonylglycitin (*r* = 0.87 ***) and genistin and malonylgenistin (*r* = 0.90 ***) (Figure 4). The positive correlations between individual isoflavone content can be attributed to their synthesis through the phenylpropanoid pathway [34]. The individual and total isoflavone content correlations agree with previous studies [8,9].

**Figure 5:**
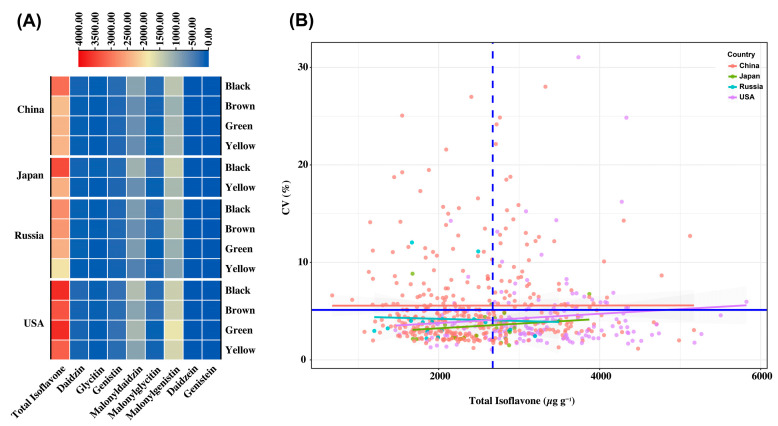
(**A**) Heatmap of individual and total isoflavones based on the origin (China, Japan, Russia, and the USA) and seed coat colors (black, brown, green, and yellow). Isoflavones correspond to the coat color and origin represented by the scale bar; the blue color on the scale bar indicates lower levels of the isoflavones and vice versa. (**B**) A scatter plot representing the correlation between the means of TIF content and the 593 soybean accessions’ coefficients of variation (CVs). The horizontal blue line shows the CV, and the mean of the TIF content is indicated by the vertical blue dashed line.

The heatmap analysis showed the individual and total isoflavone concentrations based on the country of origin and seed coat colors. Among all countries, USA soybean accessions showed higher levels of total isoflavone, malonyldaidzin, and malonylgenistin contents (Figure 5A). Among all the seed coat colors, black coat color soybean seeds showed higher levels of total isoflavone across all the countries except Russia. Interestingly, from the heatmap, it is also evident that besides black seed coat soybean accessions, green seed coat color accessions from the USA also had higher levels of total isoflavones. Because of the potentially beneficial phytochemicals in their seed coat, black soybeans have traditionally been used in folk medicine treatments in Asian nations, including China, Japan, and Korea [35,36]. Our results suggest that black coat color soybeans are reliable resources for producing higher levels of isoflavones, which can be used in food and industry to enhance human nutrition and health. However, green coat color soybean accessions from the USA can also be considered when developing soybean accessions with improved isoflavones.

Significant differences exist in the stability of the soybean accessions depends on environmental factors [37] and the MG of the accessions, as some MGs are likely to be more favored in some environments. Since a lower coefficient of variation (CV) for a cultivar in a given environment indicates more stability, the total isoflavone’s CV was utilized to reflect its stability [38,39]. To illustrate how stable the total isoflavone was within each germplasm origin, the means of the total isoflavone were plotted against their CVs (Figure 5B). Accessions with higher TIF content and low CV are desirable, as they are more stable across different environments. The soybean accessions with higher levels of total isoflavone (TIF) content and low CVs are presented in Table 2. The identified soybean accessions had isoflavone contents ranging from 4516.07 μg g^−1^ to 5823.29, with CVs ranging from 1.76 to 5.96%. Among the identified soybean accessions, 8 accessions belong to the USA, and 4 are from China. The findings suggest that introducing new germplasms to China will provide important germplasm resources with distinct isoflavone profiles, contingent upon their stability under varying environments [40]. Notably, the germplasm accessions that are highly suitable for the unique agro-environmental conditions of an area may have beneficial genetic variants and may be used in soybean breeding programs to attain acceptable isoflavone profiles, particularly the accessions from the USA.

## 3. Materials and Methods

### 3.1. Planting Materials

The Chinese Gene Bank currently preserves a collection of 28,580 soybean accessions. A subset of 2794 accessions was selected from the entire collection, which accounts for 11.8% of the total collection and contains 73.6% of its genetic diversity [11]. In our study, we utilized 593 core collection accessions, representing 21.2% of the total core collection. The accessions were selected based on their availability from the soybean genetic resource research group at the Institute of Crop Sciences (ICS), Chinese Academy of Agricultural Sciences (CAAS). These accessions are divided into different groups based on their country of origin, seed coat color, and maturity group. Based on the country of origin, they were divided into four groups: China (391 accessions), Japan (22 accessions), Russia (19 accessions), and the USA (161 accessions). The three main Chinese soybean-growing regions—the Northern, Huang Huai Hai, and Southern regions—which span an area from 23° N to 51° N, were the primary sources of the Chinese collection [31,41]. The ranges of maturity groups are MG000–II for the Northern region, II–V for the Huang Huai Hai region, and IV–VIII for the Southern region [42]. Regarding the USA collection, 161 soybean accessions were selected, including a range of maturity groups from MG000 to VIII. This selection also includes several standard varieties for maturity groups in North America [43]. The Russian and Japanese accessions used in this study were chosen as typical types from their respective countries of origin to ensure a broader range of accessions together with those from China and the USA. Soybean germplasm was divided into four categories based on their seed coat colors: yellow (478 accessions), black (59 accessions), green (30 accessions), and brown (26 accessions). Based on the maturity groups, accessions were divided into eight categories: MG0 (74 accessions), MGI (90 accessions), MGII (80 accessions), MGIII (149 accessions), MGIV (82 accessions), MGV (55 accessions), MGVI (30 accessions), and MGVII (33 accessions). The description of each soybean accession and their mean TIF contents is presented in Supplementary Table S1.

### 3.2. Field Experiments

Field experiments were conducted in Changping, Beijing, and Sanya, Hainan, in 2017 and 2018. The weather information for these locations is presented in Supplementary Table S2. The accessions were sown using randomized incomplete block design, with several planting locations as replications. Replication over locations was conducted due to the limited land resources and many accessions. The accessions were randomized independently for each location. Each accession’s seeds were sowed in 3-m rows, with inter-row and intra-row spacing set at 0.5 and 0.1 m, respectively. Nitrogen, phosphorus, and potassium were applied to the field at 30 kg ha^−1^, 40 kg ha^−1^, and 60 kg ha^−1^, respectively. From planting until maturity, standard agronomic practices were followed. Afterward, seeds from each cultivar were aggregated, and roughly 500 g of soybean seeds were taken as a sample to determine the isoflavone contents.

### 3.3. Extraction and Determination of Isoflavones

The isoflavones from soybean seeds were extracted and quantified using a methodology described by [44]. The seeds were ground into a fine powder, and 0.1 g of powder was added to a plastic tube containing 70% ethanol and 0.1% acetic acid. The mixture was shaken on a twist mixture for 12 h and centrifuged at 6000 rpm for 10 min. The supernatant was filtered through a YMC Duo filter and stored at 4 °C. Quantification was performed using an Agilent 1260 HPLC system with a YMC ODS AM-303 column. A set of 12 isoflavone standards was used to identify and quantify each component. Isoflavone concentrations were computed using the formula described by [44].

### 3.4. Statistical Analysis

Descriptive statistical analysis was performed to summarize the data corresponding to accession origin, seed coat color, and maturity groups across diverse environments. R statistical software version 3.4.5 (R Foundation for Statistical Computing, Vienna, Austria) was used for statistical analysis, encompassing analysis of variance (ANOVA), density plots, computation of Pearson’s correlation coefficients (*r*), and stability analysis. The correlation analysis was executed to uncover associations among the seed isoflavones of soybean seeds. In the ANOVA, the variable “location” (block), year and the interactions with year were designated as random effects, while the variables “accessions”, country of origin”, “seed coat color, and “maturity groups”, and their respective interactions were treated as fixed factors.

## 4. Conclusions

In the present study, we observed significant variations in seed isoflavones in soybean germplasm originating from China, Japan, Russia, and the USA, as well as seed coat color and maturity. The USA soybean accessions and black seed coat color soybean accessions showed higher levels of isoflavones. Furthermore, the late maturity group accessions had higher levels of individual and total isoflavones than early maturity groups, highlighting the significance of MG as a prominent factor affecting the composition of isoflavones in soybean seeds. Malonylglycosides were identified as the major discriminate factors among the soybean germplasms. The variations observed in isoflavone content among different soybean germplasms are due to their diverse origins, MGs, and seed coat color, which are highly important for identifying accessions with higher or lower levels of isoflavones. The highly stable soybean accessions with higher levels of TIF content will be helpful in the development of soybean varieties with enhanced nutritional profiles and diverse applications in agriculture, food, and industry. However, the stability and isoflavones content of the soybean accessions may be affected if grown at different latitudes.

## Figures and Tables

**Table 1:** Isoflavone variations in soybean germplasm of diverse origins.

Isoflavones	Range	Minimum	Maximum	Mean	Std.	CV
		(μg g^−1^)	(μg g^−1^)	(μg g^−1^)	Deviation	(%)
Daidzin	373.36	21.57	394.94	131.09	56.00	42.72
Glycitin	190.45	15.37	205.83	67.07	28.05	41.82
Genistin	468.00	41.48	509.49	221.91	78.69	35.46
Malonyldaidzin	2098.51	179.07	2277.58	771.49	326.18	42.28
Malonylglycitin	528.61	36.71	565.32	149.70	65.88	44.01
Malonylgenistin	2428.54	364.72	2793.27	1296.36	410.69	31.68
Daidzein	29.30	4.05	33.36	10.40	3.94	37.88
Genistein	26.11	4.38	30.49	12.26	3.98	32.46
Total Isoflavone	5146.04	677.25	5823.29	2670.70	857.32	32.10

**Table 2:** Accessions with higher levels of the total isoflavone (TIF) content and lower CV.

ID	TIF	CV (%)	Coat Color	Country	MG
WDD01618	5823.29	5.96	Yellow	USA	V
WDD01632	5503.4	4.57	Yellow	USA	VI
WDD00698	5263.62	2.65	Yellow	USA	V
WDD03039	5177.78	1.76	Yellow	USA	V
ZDD02450	5170.84	3.05	Black	China	I
Zhonghuang 68	4985.98	1.99	Yellow	China	III
WDD01619	4735.96	2.71	Yellow	USA	V
ZDD10734	4708.28	3.65	Black	China	VI
WDD02107	4705.91	3.63	Yellow	USA	I
ZDD02864	4671.88	3.85	Yellow	China	V
WDD03084	4649.65	2.56	Yellow	USA	II
WDD03006	4516.07	1.95	Yellow	USA	III

## Data Availability

The original contributions presented in the study are included in the article/Supplementary Material, further inquiries can be directed to the corresponding author/s.

## Supplementary Materials

The following supporting information can be downloaded at: https://www.mdpi.com/article/10.3390/plants13131774/s1, Table S1: Description and mean total isoflavone (TIF) content of soybean accessions; Table S2: Monthly temperature and precipitation readings at the experimental regions in China in 2017 and 2018; Table S3: Analysis of variance for the effects of Accession, country of origin, seed color and maturity groups on soybean seed isoflavones grown at wo locations of China for two years (separate ANOVA was performed for accession, country of origin, seed color and maturity groups [MG]); Table S4: Soybean accessions with High and low total isoflavone (TIF).
