# Supplementary material for: Effect of Origin, Seed Coat Color, and Maturity Group on Seed Isoflavones in Diverse Soybean Germplasm
Source: Plants (Basel). 2024 Jun 27;13(13):1774. doi: 10.3390/plants13131774 (PMC11243943; doi:10.3390/plants13131774)
Supplement: Supplementary file 1 [file plants-13-01774-s001.zip › plants-3061030-supplementary/Supplementary data/Supplementary material.pdf]

Supplementary Table S2 Monthly temperature and precipitation readings at the experimental regions in China in 2017 and 2018

**Sanya, Hainan Province**												
**Month**	**October**		**November**		**December**		**January**		**February**		**March**	
**Year**	**2017**	**2018**	**2017**	**2018**	**2017**	**2018**	**2017**	**2018**	**2017**	**2018**	**2017**	**2018**
**Max. (°C)**	29.0	28.0	28.0	24.0	25.0	22.0	24.0	23.0	24.0	24.0	27.0	34.0
**Aver. (°C)**	28.0	24.0	26.0	21.0	23.0	18.0	22.0	19.0	22.0	19.0	25.0	28.0
**Min. (°C)**	25.0	21.0	23.0	20.0	20.0	16.0	19	16.0	19.0	15.0	22.0	24.0
**Rainfall(mm)**	262.7	95.4	79.3	79.8	21.8	18.6	40.1	16.2	33.6	16.0	124.9	22.3
**Changping, Beijing**												
**Month**	**June**		**July**		**August**		**September**		**October**		**November**	
**Year**	**2017**	**2018**	**2017**	**2018**	**2017**	**2018**	**2017**	**2018**	**2017**	**2018**	**2017**	**2018**
**Max. (°C)**	30.0	33.0	37.0	33.0	36.0	32.0	32.0	26.0	20.0	21.0	11.0	14.0
**Aver. (°C)**	28.0	28.0	35.0	29.0	34.0	28.0	29.0	21.0	18.0	19.0	9.0	11.0
**Min. (°C)**	23.0	22.0	31.0	25.0	30.0	24.0	24.0	17.0	15.0	15.0	6.0	8.0
**Rainfall(mm)**	67.0	35.5	69.5	127	135.8	57.2	8.2	19.0	84.4	3.7	0.0	2.7

Supplementary Table S3 Analysis of variance for the effects of Accession, country of origin, seed color and maturity groups on soybean seed isoflavones grown at wo locations of China for two years (separate ANOVA was performed for accession, country of origin, seed color and maturity groups [MG])

SOV	Daidzin	Glycitin	Genistin	Malonyldaidzin	Malonylglycitin	Malonylgenistin	Daidzein	Genistein	Total Isoflavone
Accession	***	***	***	***	***	***	***	***	***
Year	***	***	**	***	***	***	*	***	***
Accession*year	***	*	*	***	***	***	NS	NS	***
Country of origin	***	***	***	***	***	***	NS	***	***
year	***	***	***	***	***	***	NS	***	***
Country of origin*year	***	NS	***	***	NS	***	*	***	***
Seed color	***	***	***	***	***	***	***	***	***
Year	***	***	***	***	***	***	NS	***	***
Seed color*year	NS	NS	NS	**	***	NS	NS	NS	NS
MG	***	***	***	***	***	***	***	***	***
Year	***	***	NS	***	***	***	NS	***	***
MG*year	NS	NS	***	NS	NS	*	NS	***	NS

*, ** and *** represent the significance levels at $p < 0.05$, 0.01, and 0.001, respectively; NS = not significant; MG = maturity group.

Table S4 Soybean accessions with High and low total isoflavone (TIF).

ID	High TIF (μg g^{-1})	Seed color	Country	Maturity group
WDD01618	5823.29	Yellow	USA	V
WDD01632	5503.40	Yellow	USA	VI
WDD00698	5263.62	Yellow	USA	V
WDD03039	5177.78	Yellow	USA	V
ZDD02450	5170.84	Black	China	I
ZDD09832	5122.67	Yellow	China	VI
Zhonghuang 68	4985.98	Yellow	China	III
WDD01974	4770.04	Yellow	USA	II
ZDD21178	4769.61	Black	China	V
WDD01619	4735.96	Yellow	USA	V
ZDD10734	4708.28	Black	China	VI
WDD02107	4705.91	Yellow	USA	I
ZDD02864	4671.88	Yellow	China	V
WDD03084	4649.65	Yellow	USA	II
WDD03006	4516.07	Yellow	USA	III

Low TIF (μg g^{-1})

ID	TIF (μg g^{-1})	Seed color	Country	Maturity group
ZDD24713	677.25	Yellow	China	IV
ZDD13152	925.49	Green	China	III
